# Defining “Phthalates”

**DOI:** 10.1289/ehp.1205763

**Published:** 2012-11-01

**Authors:** William Dale Carter

**Affiliations:** International Pharmaceutical Excipients Council of the Americas, Arlington, Virginia, E-mail: dale.carter@huber.com

The International Pharmaceutical Excipients Council of the Americas (IPEC-Americas)reviewed the article “Identification of Phthalates in Medications and Dietary Supplement Formulations in the United States and Canada” by Kelley et al. (2012). This article contains inaccuracies and misleading statements, and the terminology used by Kelly et al. was incorrect. Speaking for IPEC-Americas, I would like to rectify any confusion caused by their colloquial use of the term “phthalate.”

The term “phthalate” has been defined by the U.S. Environmental Protection Agency (EPA) and other regulatory agencies to identify diesters of orthophthalic acid, also called simply phthalic acid, an aromatic dicarboxylic acid in which the two carboxylic acid groups are located on adjacent carbons (positions 1 and 2) in the benzene ring. Both di-*n*-butyl phthalate (DBP) and di-(2-ethylhexyl) phthalate (DEHP) are examples of such phthalates; these phthalates are chemically and toxicologically distinct from diesters of isophthalic or terephthalic acids, which are not considered to be true “phthalates,” as defined by the U.S. EPA (2012). Kelley et al. (2012) failed to acknowledge these important distinctions and incorrectly grouped isophthalic and terephthalic acid derivatives with the *ortho*-phthalates. This colloquial use of “phthalates” has created unsubstantiated and erroneous safety concerns. The specific toxicological concern with DEHP and DBP arises from their metabolic conversion to their corresponding monoesters.

Kelley et al. (2012) inappropriately referred to three polymers [polyvinyl acetate phthalate (PVAP), hypromellose phthalate (HMP), and cellulose acetate phthalate (CAP)] as “phthalates” and inappropriately implied that they are “phthalates” simply because they have the word “phthalate” in their names. HMP, PVAP, and CAP are polymers that have been modified by esterification with orthophthalic acid groups. These high-molecular-weight polymers differ markedly from the short-chain alcohols used to produce DEHP and DBP, and their chemical properties are very different.

For example, HMP is an enteric polymer manufactured from the esterification of hypromellose with phthalic anhydride. HMP is a large molecule with a typical number average molecular weight in the range of 80,000–130,000 Da. DBP and DEHP are small molecules with molecular weights of only 278 and 390 Da, respectively. HMP is thus very different from DBP and DEHP, based on properties such as chemical structure and molecular weight, and has completely different functions. HMP is an enteric polymer used in pharmaceutical coatings to allow drug dissolution to take place in the intestine instead of in the stomach. DBP and DEHP are plasticizers.

Safety assessments of the enteric polymers have been performed, including acute and subacute toxicity, teratogenicity, and ADME (absorption, distribution, metabolism, and excretion) studies (Rowe et al. 2012). One published report on the long-term use of CAP indicated that no adverse findings were observed (Hodge 1944). The safety of PVAP has also been evaluated in a definitive 90-day subchronic toxicity study, a developmental toxicity study, and several genotoxicity tests; however, the results have not yet been published (DeMerlis CC, unpublished data). No adverse effects were reported in either the 90-day subchronic toxicity study or the developmental toxicity study, and PVAP was not genotoxic.

The toxicity associated with DBP and DEHP stems from their bioconversion to their respective monoesters; this bioconversion is unlikely for PVAP and not possible for HMP or CAP. These distinctions are further affirmed by the draft guidance for limiting the use of specific phthalates as excipients (Food and Drug Administration 2012), which applies only to DBP and DEHP and does not include either HMP, CAP, or PVAP.

In conclusion, it is inappropriate to use a common name to incriminate a group of structurally diverse compounds simply because they share one common structural feature. This practice has created unsubstantiated safety concerns where none exist.
